# Broadband Perfect Optical Absorption by Coupled Semiconductor Resonator-Based All-Dielectric Metasurface

**DOI:** 10.3390/ma12081221

**Published:** 2019-04-14

**Authors:** Zhi Weng, Yunsheng Guo

**Affiliations:** 1 College of Electronic Information Engineering, Inner Mongolia University, Hohhot 010021, China; wzhi@imu.edu.cn; 2 School of Physical Science and Technology, Inner Mongolia University, Hohhot 010021, China; 3 Department of Applied Physics, Inner Mongolia University of Science and Technology, Baotou 014010, China

**Keywords:** perfect absorption, optical broadband, semiconductor metasurface, coupled resonators, optical device

## Abstract

Resonance absorption mechanism-based metasurface absorbers can realize perfect optical absorption. Further, all-dielectric metasurface absorbers have more extensive applicability than metasurface absorbers that contain metal components. However, the absorption peaks of the all-dielectric metasurface absorbers reported to date are very sharp. In this work, we propose a broadband optical absorption all-dielectric metasurface, where a unit cell of this metasurface is composed of two coupled subwavelength semiconductor resonators arrayed in the direction of the wave vector and embedded in a low-index material. The results indicate that the peak absorption for more than 99% is achieved across a 60 nm bandwidth in the short-wavelength infrared region. This absorption bandwidth is three times that of a metasurface based on the conventional design scheme that consists of only a single layer of semiconductor resonators. Additionally, the coupled semiconductor resonator-based all-dielectric metasurface shows robust perfect absorption properties when the geometrical and material parameters—including the diameter, height, permittivity, and loss tangent of the resonator and the vertical and horizontal distances between the two centers of the coupled resonators—are varied over a wide range. With the convenience of use of existing semiconductor technologies in micro/nano-processing of the surface, this proposed broadband absorption all-dielectric metasurface offers a path toward realizing potential applications in numerous optical devices.

## 1. Introduction

Optical absorption is a typical problem in light-matter interactions. Strengthening and controlling optical absorption has long been a significant research topic in the fields of optics and optoelectronics. Perfect light absorption, which implies zero reflection, zero transmission, and maximum absorption, has many potential applications in devices such as absorbers [1,2,3,4], sensors [5,6,7], photodetectors [8,9], thermal emitters [10,11], and energy harvesting devices [12,13,14,15]. Therefore, the exploration of the many different types of perfect absorbing mechanism is extraordinarily important. An electromagnetic metasurface, which is composed of a flat plate array of subwavelength resonators [16,17,18], has a very flexible construction approach and can thus realize many unconventional electromagnetic parameters when interacting with external electromagnetic waves. By adjusting both the equivalent relative permittivity and permeability of the metasurface to have identical values within a certain frequency band, the characteristic impedance of the metasurface will then be well matched with that of a vacuum, and thus a plate with zero reflection is formed [19]. Additionally, by selecting appropriate values for the electromagnetic loss parameters of the materials used in the metasurface, the incident wave energy may be dissipated completely and thus a plate with zero transmission can also be obtained [20]. When zero reflection and zero transmission characteristics are presented simultaneously in the same plate, perfect absorption is realized. At present, perfect absorption metasurfaces are mainly embodied using two types of construction schemes: one uses metal-dielectric mixed structures [1,2,3,4,5,6,7,8,9,10,11,12,13,14,15] and the other uses all-dielectric structures [21,22,23]. Because of the high ohmic losses, low melting points, and high thermal conductivities of the metals [21], the applicability of metal-dielectric metasurface-based perfect absorption is decidedly limited in some cases. All-dielectric metasurfaces can make up for the shortcomings of the metal-dielectric metasurfaces and have much wider application prospects. In recent years, perfect absorption in all-dielectric metasurfaces operating in the terahertz regime has been demonstrated theoretically using crystalline quartz resonators [22] and has also been demonstrated experimentally using silicon resonators [21]. This is a critical step toward unlocking the potential applications of these metasurfaces in devices such as terahertz absorbers and detectors. Furthermore, the absorption frequency can be tuned by varying the geometrical parameters of the resonators [23]. Therefore, it appears that all-dielectric metasurface-based perfect absorption at different frequencies can be resolved. However, the perfect absorption bandwidths of the reported all-dielectric metasurfaces are all extremely narrow [21,22,23]. Therefore, exploration of broad absorption band all-dielectric metasurfaces is of major importance. 

In this work, we first constructet an all-dielectric metasurface absorber based on the conventional absorption mechanism. The unit cell structure was simply a subwavelength silicon nanodisk resonator surrounded by a low-index silicon dioxide bulk. By tuning the silicon disk geometry and overlapping the electric and magnetic dipole resonances at the same frequency, perfect narrowband optical absorption was achieved. Because this narrowband characteristic was caused by the sharp transmission valley, we placed another silicon nanodisk into the unit cell and built a coupling system composed of two silicon resonators in the direction of wave propagation. By adjusting the coupling distance between these two resonators and selecting an optimal distance value, a broad transmission valley was realized and a broad absorption band with a center wavelength of 1.3 μm and a 60 nm near-perfect absorption bandwidth was obtained. In contrast, a near-perfect absorption bandwidth of only 20 nm was achieved in the single silicon resonator-built metasurface. More importantly, when parameters such as the height, diameter, permittivity, and loss tangent of the two coupled resonators and the vertical and horizontal distances between them are varied over a wide range, the unit cell still demonstrates robust and perfect wideband absorption features. Therefore, the proposed metal-free metasurface based on coupled semiconductor resonators will pave the way toward applicable optical devices.

## 2. Theory, Models, and Results

### 2.1. Narrowband Perfect Optical Absorption All-Dielectric Metasurface

In recent years, numerous experiments have demonstrated that the subwavelength high-index particles that are used as unit cells can also be used to construct all-dielectric metasurfaces that operate not only in the microwave [24,25] and THz [26,27] bands but in the infrared [28,29] and visible [30,31] bands. This is possible because of the strong resonant response that occurs when external electromagnetic waves interact with the subwavelength particles, which are called resonators. While multiple resonant responses are induced in these dielectric particles, only the electric and magnetic dipole resonances are currently widely adopted in metasurfaces because electricity and magnetism represent the basic properties of these materials. The resonant frequencies of the magnetic and electric dipoles are related to the geometrical and material parameters of the particles. Generally, when an electric resonance or magnetic resonance occurs in the dielectric particles, almost all the incident waves are reflected, and the transmitted waves are close to zero. However, when the electric and magnetic dipole resonances emerge simultaneously at the same frequency, the equivalent permittivity and permeability of the dielectric particles are then equal, and thus the characteristic impedance of the particle unit cell is matched with that of the vacuum. In such a case, zero reflectance for all incident waves is achieved. To realize perfect absorption by these particles, one needs to reduce the transmitted wave to zero. It is known that most of the incident electromagnetic waves are concentrated in the particles in the resonant state [32], which means that a moderate dielectric loss can completely dissipate the strongly localized electromagnetic energy in the particles and thus zero transmittance can also be achieved. In semiconductor materials, the dielectric loss can easily be adjusted by doping the semiconductor with boron or aluminum [33,34]. In addition, most semiconductor materials, such as silicon and silicon carbide, have relatively high permittivities in the optical band. Therefore, the use of semiconductor particles to build an all-dielectric metasurface with perfect optical absorption is a viable alternative solution.

The disk height and diameter can be adjusted independently. The disk-shaped subwavelength nanoparticle is the preferred unit cell for metasurface design. The height and diameter of the disk for the disk unit cell of the all-dielectric metasurface can be determined from the permittivity of the disk and the resonant operating wavelength, as shown in the following formulae [21]:

(1) $$h=\frac{\lambda_{0}}{2\sqrt{\varepsilon_{r}}}$$

(2) $$d=\frac{3.83\lambda_{0}}{\pi \sqrt{\varepsilon_{r}-1}}$$

where *h* and *d* are the height and the diameter of the disk, respectively; *λ*_0_ is the operating wavelength of the light in a vacuum; and *ε_r_* is the permittivity of the semiconductor material used to form the disk. Therefore, when the operating wavelength *λ*_0_ and the permittivity *ε_r_* are determined, the height and diameter of the disk can then be calculated roughly using Equations (1) and (2). It should be noted here that Equations (1) and (2) are both based on the condition that the disk is surrounded by a vacuum. During fabrication of the metasurfaces, a substrate and an embedding medium must be added; therefore, they should be considered first in the design process. In addition, many simulation software packages, such as CST 2011, provide powerful tools for metasurface design. Therefore, an all-dielectric metasurface can be realized precisely in advance using a combination of theoretical calculations and electromagnetic simulations.

**Figure 1:**
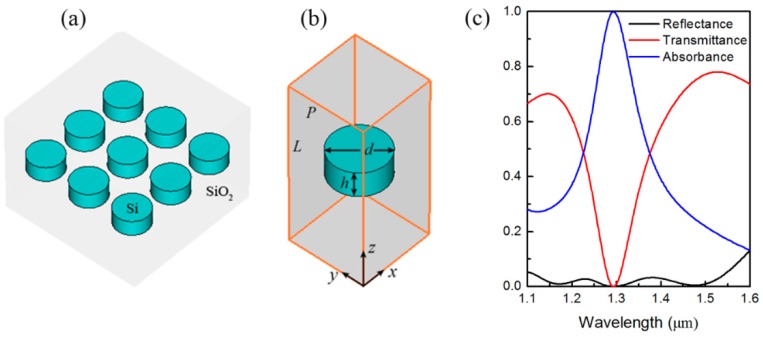
Schematic representation of narrowband all-dielectric metasurface composed of only a single layer of subwavelength semiconductor resonators and its spectral properties. (**a**) Periodic array structure composed of silicon nanodisks embedded into silicon dioxide. (**b**) Unit cell structure with its geometric parameters indicated, where *h* = 220 nm, *d* = 470 nm, *L* = 1440 nm, and *P* = 670 nm. (**c**) Numerically calculated reflectance (black line), transmittance (red line), and absorbance (blue line) spectra.

Figure 1 shows a schematic representation of the simulated all-dielectric metasurface structure and its spectral properties. The periodic array and the unit cell of the structure can be seen in Figure 1a,b, respectively. The unit cell consists of a silicon disk that is embedded in the center of the silicon dioxide medium. The relative permittivities of silicon and silicon dioxide are 12.25 and 2.25, respectively. We want to design a perfect absorption all-dielectric metasurface for operation in the near infrared band; the height of the disk is thus set at *h* = 220 nm based on the reported experimental results [19], and the height of the silicon dioxide layer is *L*= 1440 nm. The disk diameter is set as a variable and the lattice constant *P* is varied accordingly, together with the diameter *d*, where *P* = *d* + 200 nm. The disk diameter is optimized at *d* = 470 nm to ensure that the electric and magnetic dipole resonances within the silicon disk overlap; this occurs when the unit cell is assumed to be lossless and the scattering parameters |S_21_| and |S_11_| approach unity and zero at the operating wavelength, respectively. All simulations in this work were performed using CST 2011 with periodic boundary conditions enabled in the *x*- and *y*-directions and open (add space) boundary conditions enabled in the *z*-direction. The optical signal was simulated using a default plane wave at normal incidence on the structure in the frequency domain solver. An adaptively refined tetrahedral mesh was then applied to the simulated structure with an average size of 20,000 cells. The absorbance *A*(*λ*_0_) is strongly dependent on the transmittance *T*(*λ*_0_) and the reflectance *R*(*λ*_0_), and the relationship between these parameters is *A*(*λ*_0_) = 1 − *T*(*λ*_0_)−*R*(*λ*_0_), where *T*(*λ*_0_) = |*S*_21_|^2^, and *R*(*λ*_0_) = |*S*_11_|^2^. The scattering parameters |*S*_21_| and |*S*_11_| can be obtained directly from the simulation results. Therefore, the absorbance *A*(*λ*_0_), the transmittance *T*(*λ*_0_), and the reflectance *R*(*λ*_0_) can easily be worked out. Figure 1c shows the calculated spectral dependences of *R*(*λ*_0_), *T*(*λ*_0_), and *A*(*λ*_0_) over the wavelength range from 1.1 μm to 1.6 μm. We can clearly see that *R*(*λ*_0_) is less than 0.1 over a wide wavelength range from 1.1 μm to 1.55 μm and zero reflection occurs at a wavelength of 1.3 μm. However, unlike *R*(*λ*_0_), *T*(*λ*_0_) shows only a very sharp zero transmission valley at 1.3 μm and its value increases rapidly as the operating wavelength gradually moves away from 1.3 μm. Therefore, an all-dielectric metasurface based on a single-layer of subwavelength silicon resonators with a near-perfect absorption (*A*(*λ*_0_) > 0.99) band across the wavelength range from 1.29 μm to 1.31 μm is obtained.

### 2.2. Broadband Perfect Optical Absorption All-Dielectric Metasurface

**Figure 2:**
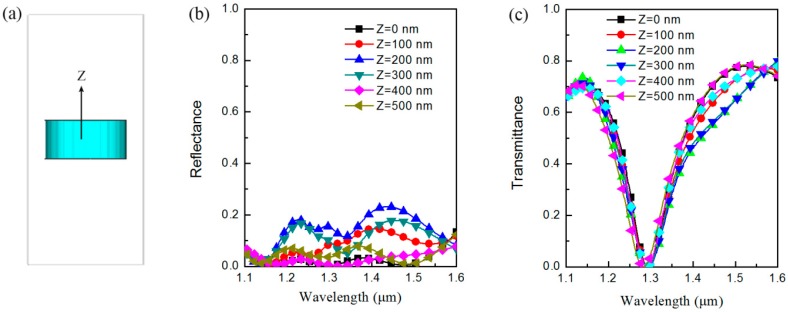
Silicon disk position and its *z*-direction-dependent reflectance and transmittance characteristics. (**a**) Schematic representation of the silicon disk at the center of the unit cell and the direction of silicon movement. (**b**) Reflectance and (**c**) transmittance characteristics of the unit cell when the silicon disk position varies from *z* = 0 to *z* = 500 nm in steps of 100 nm.

Figure 1 illustrates the structure and the mechanism of the perfect optical absorption all-dielectric metasurface, but the perfect absorption band is very narrow. Figure 1c clearly shows that the narrow absorption band is mainly induced by the sharp transmittance valley because the reflection is maintained at nearly zero over a wide wavelength range. Therefore, to realize a broadband absorption metasurface, one important problem that must be solved is the broadening of this transmission valley. Generally speaking, coupling two resonators in the direction of wave propagation can efficiently broaden the transmission bandwidth. For example, dual-band-enhanced transmission using coupled metamaterial resonators [35], total broadband transmission by localized E-field coupling of split-ring resonators [36], and Mie-resonance-coupled total broadband transmission [37] have all been reported in our previous work. In this work, we intend to realize a broadband perfect absorption all-dielectric metasurface using two coupled resonators. However, before the second silicon disk is added to the unit cell of the metasurface, the effect of the silicon disk position in the *z*-direction in the unit cell on the reflectance and transmittance characteristics should be considered. Figure 2 shows the simulated reflectance of *R*(*λ*_0_) and transmittance *T*(*λ*_0_) characteristics when the silicon disk is shifted from the center of the unit cell (*z* = 0) to a position of *z* = 500 nm. We see from Figure 2b that *R*(*λ*_0_) does indeed change with the position of the silicon disk, but its amplitude is relatively small. For example, when the silicon disk moves by a distance of 400 nm, the changes in *R*(*λ*_0_) can be regarded as negligible. In the other movement distance cases, *R*(*λ*_0_) is always maintained at values of less than 0.2. With regard to the effect of the movement distance of the silicon disk on *T*(*λ*_0_), Figure 2c clearly shows that the position of the silicon disk has no obvious influence on the transmitted waves, particularly in the region near the sharp transmission valley. If we select an optimized moving distance, e.g., *z* = 400 nm, a perfect absorption metasurface with an asymmetric unit cell structure in the *z*-direction is realized. It should be noted that this asymmetric structure changes the ratios of the electric and magnetic resonances within the silicon disk and introduces a slight bianisotropy. Therefore, perfect absorption is only achieved for light waves that are incidental from one specific side, although the transmission is zero in both directions because of reciprocity.

**Figure 3:**
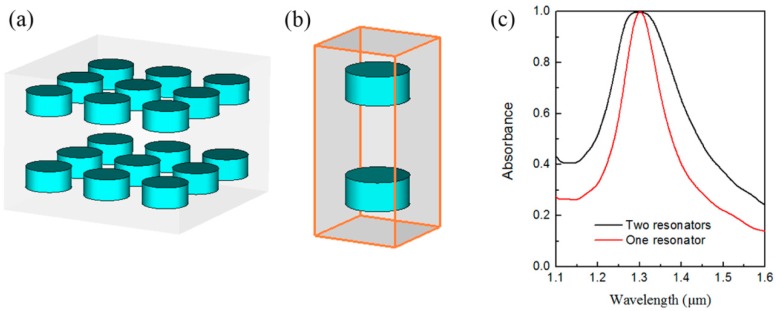
Schematic representation of the proposed broadband all-dielectric metasurface structure and its absorbance spectrum. (**a**) Periodic array and (**b**) unit cell structure of the metasurface, where the unit cell is composed of two coupled silicon disks embedded in silicon dioxide. (**c**) Numerically calculated absorbance spectra of the double resonator unit cell (black line) and the single resonator unit cell (red line), where the latter is provided for comparison.

Because the optimized silicon disk position does not affect the perfect absorption property of the structure, we can introduce another silicon disk into the unit cell and place it symmetrically in the opposite *z*-direction. The above analyses indicate that this two-coupled-resonators unit cell-based all-dielectric metasurface can not only realize a symmetrical absorption structure but also provides a broadband absorption structure. Figure 3a shows a schematic representation of the proposed metasurface structure, which consists of two layers of coupled resonators; the distance between these layers is 800 nm. The geometrical parameters of the unit cell shown in Figure 3b are the same as the parameters that were depicted in Figure 1b. The numerically calculated absorbance characteristics of the two-coupled-resonators-based all-dielectric metasurface are shown in Figure 3c. For contrast, the absorbance of the metasurface composed of only a single silicon resonator layer is also shown in Figure 3c. The figure clearly shows that a near-perfect absorption (*A*(*λ*_0_) > 0.99) band is formed across the wavelength range from 1.27 μm to 1.33 μm, which represents a 60 nm bandwidth and is three times the size of the corresponding band for the metasurface composed of unit cells containing only single silicon resonators. In addition to the absorption band at *A*(*λ*_0_) > 0.99, the absorption band at any other absorbance value of the metasurface composed of two coupled resonators is approximately twice as broad as that for the metasurface composed of single resonator unit cells. Therefore, using these two coupled resonators, in which the electric and magnetic dipole resonances form simultaneously at the same wavelength, a broadband optical absorption all-dielectric metasurface can be realized.

**Figure 4:**
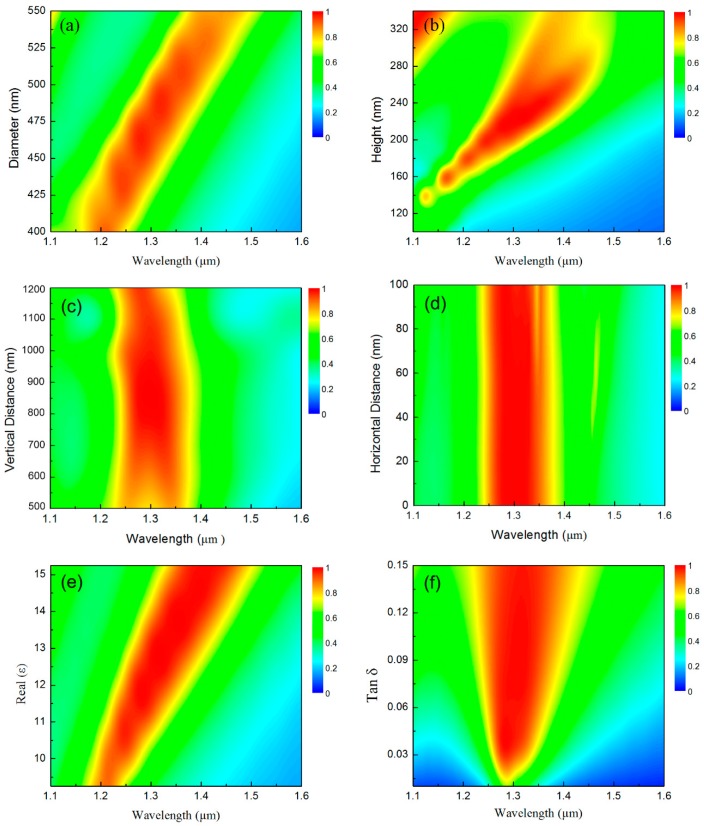
Robust perfect absorption properties of the proposed broadband all-dielectric metasurface. The dependence of the absorbance on the operating wavelength are on: (**a**) the diameter; (**b**) the height of the silicon disks; (**c**) the vertical and (**d**) the horizontal distances between the centers of the two silicon disks; (**e**) the real part of the permittivity; and (**f**) the loss tangent of the silicon disks.

To demonstrate the robust perfect absorption properties of the proposed broadband all-dielectric metasurface made from coupled semiconductor resonator pairs, Figure 4a–f show color maps of the spectral absorbance as a function of the operating wavelength from 1.1 μm to 1.6 μm and the dependence on the geometrical and material parameters of the silicon disks. Figure 4a shows the calculated results for the absorption spectrum when the diameter of the silicon disks varies from 400 nm to 550 nm, while all other parameters remain fixed. We observe that while the absorption peak shifts toward longer wavelengths as the diameter increases, the absorbance and bandwidth show almost no change. Figure 4b illustrates the absorption spectrum as the height of the silicon disks varies from 100 nm to 340 nm. Similarly, we see that the absorbance and the bandwidth also maintain steady values over a wider height range from 200 nm to 260 nm. Figure 4c shows the absorption spectrum when the vertical distance between the centers of the two silicon disks varies from 500 nm to 1200 nm. We see that the bandwidth decreases gradually as the vertical distance exceeds 1000 nm and the absorption band splitting and damping arises when the vertical distance is less than 650 nm. The absorbance and bandwidth remain stable when the vertical distance varies from 650 nm to 1000 nm. These phenomena are caused by the coupling state (under-coupling, critical coupling, and over-coupling) of the two resonators, which is determined by the coupling distance [38]. 

Figure 4d illustrates the absorption spectrum as the horizontal distance between the centers of the two silicon disks varies from 0 to 100 nm. We do not see any change except that a very narrow parasitic band with low absorbance (*A*(*λ*_0_) < 0.8) appears in the perfect absorption band when the horizontal distance is larger than 50 nm. In addition to the geometrical parameters, we also studied the spectral absorption as a function of the material parameters, including the real part of the permittivity and the loss tangent of the disks; these final calculated absorbance spectra are shown in Figure 4e,f, respectively. These figures clearly show that the absorbance and bandwidth remain stable when the real part of the permittivity is no more than 11 or the loss tangent of the disks is no more than 0.03. Furthermore, as the real part of the permittivity and the loss tangent of the silicon disks increase, the metasurface presents increasingly excellent absorption performances. 

**Figure 5:**
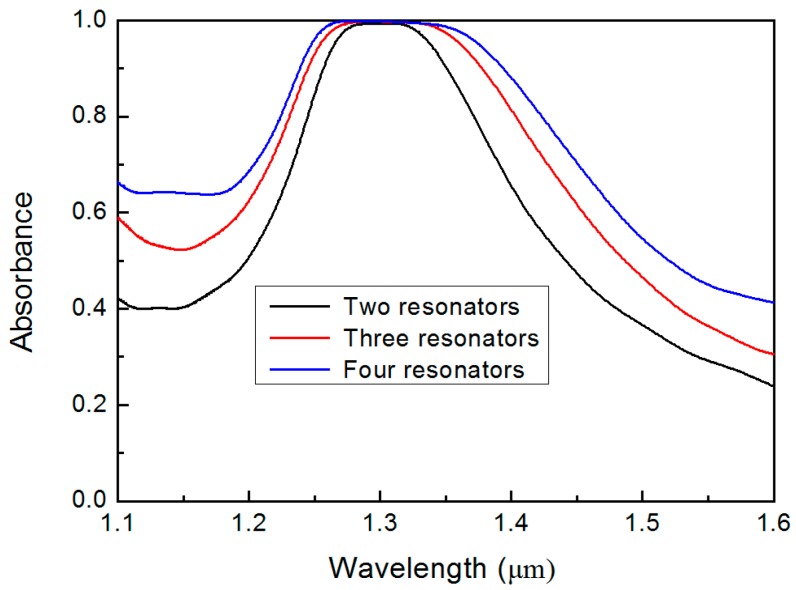
Numerically calculated absorbance spectra of the two (black line), three (red line), and four (blue line) coupled resonators in the unit cell of the proposed all-dielectric metasurface.

Overall, the proposed broadband absorption all-dielectric metasurface has a very simple structure and is quite robust against fabrication inaccuracies in terms of its geometrical and material parameters. Finally, we calculated the effects of the increasing number of coupled silicon disk on the absorption properties. Figure 5 shows the absorbance of the unit cell composed of two, three, and four silicon disks. We can clearly see that the perfect absorption bandwidth is significantly broadened as the number of silicon disk increases from two to four. Because the absorption bandwidth is mainly determined by the transmission valley, increasing the number of coupled resonators in the direction of wave propagation can effectively broaden the absorption bandwidth. 

It should be noted that although only simulated models of the broadband absorption are presented, the actual absorbers can be accurately processed using boron doped silicon on insulator substrate. Moreover, the measured absorption spectra showed a good agreement with the simulated ones [21]. Therefore, the design method and simulated models in this work is feasible. More information about sample processing and experimental measurement can be found in references [19,20,21].

## 3. Conclusions

In summary, we have designed a broadband optical perfect absorption all-dielectric metasurface. The unit cell of this metasurface is composed of two coupled semiconductor silicon resonators that are embedded in a low dielectric constant silicon dioxide material. The simulation results show that the proposed metasurface has near perfect absorption (*A*(*λ*_0_) > 0.99)) within a 60 nm bandwidth, which is three times the size of the corresponding bandwidth for the metasurface composed of only a single layer of silicon resonators. Furthermore, when the semiconductor resonator parameters and the distance between two coupled resonators are varied over a wide range, the metasurface maintains its excellent absorption properties in terms of both efficiency and bandwidth. Because the semiconductor technology used in micro/nano-processing is very mature, the proposed metasurface provides a feasible solution for the fabrication and application of high-performance all-dielectric optical absorbers and sensors.

## References

[1] Landy N., Sajuyigbe S., Mock J., Smith D., Padilla W. (2008). Perfect metamaterial absorber. Phys. Rev. Lett..

[2] Liu X., Starr T., Starr A., Padilla W. (2010). Infrared spatial and frequency selective metamaterial with near-unity absorbance. Phys. Rev. Lett..

[3] Guo Y., Hou X., Lv X., Bi K., Lei M., Zhou J. (2017). Tunable artificial microwave blackbodies based on metasurfaces. Opt. Express.

[4] Guo Y., Li J., Hou X., Lv X., Liang H., Zhou J. (2017). A simple topology metamaterial blackbody for visible light. J. Alloys Compd..

[5] Shrekenhamer D., Chen W., Padilla W. (2013). Liquid crystal tunable metamaterial absorber. Phys. Rev. Lett..

[6] Liu N., Mesch M., Weiss T., Hentschel M., Giessen H. (2010). Infrared perfect absorber and its application as plasmonic sensor. Nano Lett..

[7] Janneh M., De Marcellis A., Palange E., Tenggara A., Byun D. (2018). Design of a metasurface-based dual-band Terahertz perfect absorber with very high Q-factors for sensing applications. Opt. Commun..

[8] Rifat A., Rahmani M., Xu L., Miroshnichenko A. (2018). Hybrid metasurface based tunable near-perfect absorber and plasmonic sensor. Materials.

[9] Li W., Valentine J. (2014). Metamaterial perfect absorber based hot electron photodetection. Nano Lett..

[10] Liu X., Tyler T., Starr T., Starr A., Jokerst N., Padilla W. (2011). Taming the blackbody with infrared metamaterials as selective thermal emitters. Phys. Rev. Lett..

[11] Feng R., Ding W., Liu L., Chen L., Qiu J., Chen G. (2014). Dual-band infrared perfect absorber based on asymmetric T-shaped plasmonic array. Opt. Express.

[12] Dincer F. (2015). Electromagnetic energy harvesting application based on tunable perfect metamaterial absorber. J. Electromagn. Waves Appl..

[13] Bakir M., Karaaslan M., Dincer F., Akgol O., Sabah C. (2016). Electromagnetic energy harvesting and density sensor application based on perfect metamaterial absorber. Int. J. Mod. Phys. B.

[14] Yin M., Tian X., Wu L., Li D. (2013). A broadband and omnidirectional electromagnetic wave concentrator with gradient woodpile structure. Opt. Express.

[15] Muhammad N., Fu T., Liu Q., Tang X., Deng Z., Ouyang Z. (2018). Plasmonic metasurface absorber based on electro-optic substrate for energy harvesting. Materials.

[16] Lin D., Fan P., Hasman E., Brongersma M. (2014). Dielectric gradient metasurface optical elements. Science.

[17] Yang Y., Kravchenko I.I., Briggs D., Valentine J. (2014). All-dielectric metasurface analogue of electromagnetically induced transparency. Nat. Commun..

[18] High A., Devlin R., Dibos A., Polking M., Wild D., Perczel J., de Leon N., Lukin M., Park H. (2015). Visible-frequency hyperbolic metasurface. Nature.

[19] Staude I., Miroshnichenko A., Decker M., Fofang N., Liu S., Gonzales E., Dominguez J., Luk T., Neshev D., Brener I. (2013). Tailoring directional scattering through magnetic and electric resonances in subwavelength silicon nanodisks. ACS Nano.

[20] Moitra P., Slovick B., Yu Z., Krishnamurthy S., Valentine J. (2014). Experimental demonstration of a broadband all-dielectric metamaterial perfect reflector. Appl. Phys. Lett..

[21] Liu X., Fan K., Shadrivov I., Padilla W. (2017). Experimental realization of a terahertz all-dielectric metasurface absorber. Opt. Express.

[22] Cole M., Powell D., Shadrivov I. (2016). Strong terahertz absorption in all-dielectric Huygens’ metasurfaces. Nanotechnology.

[23] Tian J., Luo H., Li Q., Pei X., Du K., Qiu M. (2018). Near-infrared super-absorbing all-dielectric metasurface based on single-layer germanium nanostructures. Laser Photonics Rev..

[24] Zhu W., Xiao F., Kang M., Premaratne M. (2016). Coherent perfect absorption in an all-dielectric metasurface. Appl. Phys. Lett..

[25] Li L., Wang J., Wang J., Du H., Huang H., Zhang J., Qu S., Xu Z. (2015). All-dielectric metamaterial frequency selective surfaces based on high-permittivity ceramic resonators. Appl. Phys. Lett..

[26] Mitrofanov O., Siday T., Thompson R., Luk T., Brener I., Reno J. (2018). Efficient photoconductive terahertz detector with all-dielectric optical metasurface. APL Phontonics.

[27] Yang Q., Chen X., Xu Q., Tian C., Xu Y., Cong L., Zhang X., Li Y., Zhang C., Zhang X. (2018). Broadband terahertz rotator with an all-dielectric metasurface. Photonics Res..

[28] Forouzmand A., Salary M., Inampudi S., Mosallaei H. (2018). A tunable multigate Indium-Tin-Oxide-assisted all-dielectric metasurface. Adv. Opt. Mater..

[29] Owiti E., Yang H., Liu P., Ominde C., Sun X. (2018). Polarization converter with controllable birefringence based on hybrid all-dielectric-graphene metasurface. Nanoscale Res. Lett..

[30] Liu S., Vabishchevich P., Vaskin A., Reno J., Keeler G., Sinclair M., Staude I., Brener I. (2018). An all-dielectric metasurface as a broadband optical frequency mixer. Nat. Commun..

[31] Huang Y., Xu H., Lu Y., Chen Y. (2018). All-dielectric metasurface for achieving perfect reflection at visible wavelengths. J. Phys. Chem. C.

[32] Guo Y., Liu S., Bi K., Lei M., Zhou J. (2018). Low-power nonlinear enhanced electromagnetic transmission of a subwavelength metallic aperture. Photonics Res..

[33] Kinsey N., DeVault C., Kim J., Ferrera M., Shalaev V., Boltasseva A. (2015). Epsilon-near-zero Al-doped ZnO for ultrafast switching at telecom wavelengths. Optica.

[34] Naik G., Shalaev V., Boltasseva A. (2013). Alternative plasmonic materials: Beyond gold and silver. Adv. Mater..

[35] Guo Y., Zhou J. (2015). Dual-band-enhanced transmission through a subwavelength aperture by coupled metamaterial resonators. Sci. Rep..

[36] Guo Y., Zhou J. (2014). Total broadband transmission of microwaves through a subwavelength aperture by localized E-field coupling of split-ring resonators. Opt. Express.

[37] Guo Y., Zhou J., Lan C., Wu H., Bi K. (2014). Mie-resonance-coupled total broadband transmission through a single subwavelength aperture. Appl. Phys. Lett..

[38] Sample A., Meyer D., Smith J. (2011). Analysis, experimental results, and range adaptation of magnetically coupled resonators for wireless power transfer. IEEE Trans. Ind. Electron..

